# Genetic engineering and the eye

**DOI:** 10.1038/s41433-024-03441-2

**Published:** 2024-11-08

**Authors:** Rory Murphy, Keith R. Martin

**Affiliations:** 1 Department of Ophthalmology, Royal Victoria Eye and Ear Hospital, Dublin, Ireland; 2 Department of Ophthalmology, Royal Victorian Eye and Ear Hospital, Melbourne, VIC Australia; 3 Ophthalmology, Department of Surgery, University of Melbourne, Melbourne, VIC Australia; 4 Centre for Eye Research Australia, Royal Victorian Eye and Ear Hospital, Melbourne, VIC Australia

**Keywords:** Personalized medicine, Eye abnormalities

## Abstract

The transformative potential of genetic engineering in ophthalmology is remarkable, promising new treatments for a wide range of blinding eye diseases. The eye is an attractive target organ for genetic engineering approaches, in part due to its relatively immune-privileged status, its accessibility, and the ease of monitoring of efficacy and safety. Consequently, the eye has been at the forefront of genetic engineering advances in recent years. The development of Clustered regularly interspaced short palindromic repeats/CRISPR-associated protein 9 (CRISPR/Cas9), base editors, prime editors, and transposases have enabled efficient and specific gene modification. Ocular gene therapy continues to progress, with recent advances in delivery systems using viral / non-viral vectors and novel promoters and enhancers. New strategies to achieve neuroprotection and neuroregeneration are evolving, including direct in-vivo cell reprogramming and optogenetic approaches. In this review, we discuss recent advances in ocular genetic engineering, examine their current therapeutic roles, and explore their potential use in future strategies to reduce the growing burden of vision loss and blindness.

## Introduction

The concepts of ‘genetic engineering’, and ‘genetically modified organisms’, have been topics of wide public interest for decades. From pop culture fictional references in Steven Spielberg’s 1993 film ‘Jurassic Park’, where dinosaurs were genetically engineered from the preserved DNA in fossilised mosquitos, to current global attempts to eradicate malaria using genetically engineered mosquitos [1, 2], or develop disease resistant crops [3], the possible applications of genetic engineering are extensive. Genetic engineering can also facilitate the creation of animal models of disease, aiding our understanding of causation and providing platforms to trial new treatments [4].

Although genetic conditions were initial targets for gene therapy, cancer has emerged as a disease area where the strategy has many potential applications. Indeed, over the past decade, the number of gene therapy trials related to cancer has been more than double the combined trials for genetic disorders and infectious diseases [5].

**Fig. 1:**
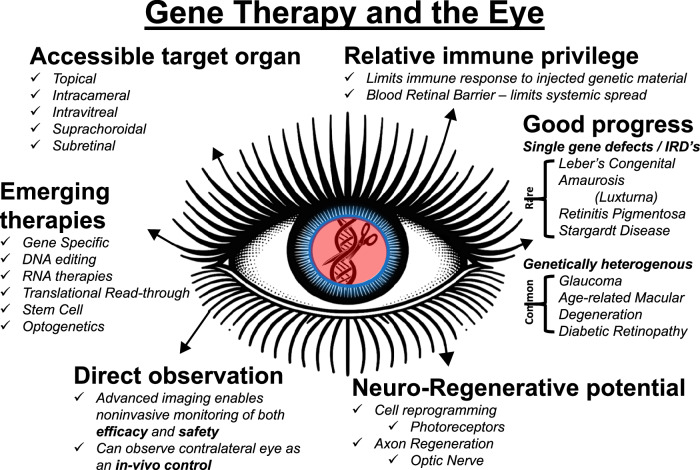
Genetic engineering and the eye; the eye as an attractive target organ, recent progress and emerging therapies. DNA Deoxyribonucleic Acid, IRD Inherited Retinal Diseases, RNA Ribonucleic acid.

Within ophthalmology, applications of gene therapy are not restricted to monogenetic and rare conditions such as Inherited Retinal Diseases (IRD). Whilst the very first FDA approved gene therapy was for *RPE65*-mediated inherited retinal dystrophy [6], evolving strategies are making the technology applicable to more genetically heterogenous common conditions such as glaucoma, diabetic retinopathy and age-related macular degeneration. (Fig. 1) Indeed, the eye is an attractive target organ for genetic engineering approaches, in part due to its relatively immune-privileged status, its accessibility, and the ease of monitoring of efficacy and safety. Consequently, the eye has been at the forefront of genetic engineering advances in recent years.

Several recent reviews have elegantly outlined numerous advances within the field of ocular gene therapy [7–11]. In the current review, we will provide some selected examples of the use of several different types of genetic engineering technology as applied to the eye.

## Genetic engineering as an engineering discipline

Genetic engineering refers to the use of molecular biology technology techniques to manipulate the structure of nucleic acids at specific locations, thus altering the genetic makeup of an organism [12]. Different strategies employing varied molecular mechanisms have been developed, with far reaching applications not only in medicine, but in agriculture, and biotechnology. Genetic engineering shares many similarities with conventional engineering principles, including the need for problem solving approaches, precision, and application of fundamental scientific principles in the design and manipulation of physical systems. As with other branches of engineering, genetic engineering requires innovation, creativity, iterative testing and refinement to achieve desired results. However, genetic engineering approaches also carry potential risks. Indeed, much of the early progress in gene therapy technology was overshadowed following the tragic death of Jesse Gelsinger, an 18 year old volunteer for a phase 1 clinical gene therapy trial for Ornithine transcarbamylase deficiency [13], and the ill-fated development of T-cell leukaemia in trial patients for gene therapy for X-linked severe combined immune deficiency (X-SCID) [14, 15]. These setbacks highlighted the need for strict safety standards and appropriate regulatory processes. In addition, there are ethical and moral dimensions to genetic engineering that potentially distinguish it from other engineering disciplines and require careful consideration. For instance, concern over the potential misuse of gene editing techniques in human embryos to enhance desirable traits came to the forefront in 2018 when Chinese twins, whose genome had been edited prior to birth to prevent HIV infection, were born [16]. This sparked fierce criticism from bioethicists and many in the global gene therapy research community, resulting in calls for a global moratorium on heritable genome editing and an international governance framework [17]. As our capabilities grow with innovative technologies, so too must our understanding of possible undesired consequences. It is important that stakeholders including doctors, researchers, ethicists and governments remain committed to prioritising safety for patients and society as new genetic technologies evolve.

## Strategies and molecular tools to alter the genome

Gene therapy can be achieved by introduction of a functional gene to replace a defective gene (gene replacement), suppression or reduction of gene expression where its expression is associated with disease (gene silencing), transfer of an exogenous gene to compensate for a missing of faulty gene (gene addition), or introducing a gene corrector which repairs a mutant gene to restore function (gene editing). Most gene editing is achieved by inducing double strand breaks (DSBs) at preselected target sites in DNA using engineered nucleases acting like molecular scissors. Available enzymes include Zinc Finger Nucleases (ZFNs) [18], Transcription Activator-Like Effector Nucleases (TALENs) [19] and Cas nucleases of the CRISPR/Cas9 system [20] which act at predetermined genomic loci by using single-guided RNA to reduce off target activity [21]. Following an induced double strand break in DNA, repair is either by non-homologous end joining (NHEJ) or homology directed repair (HDR) [22]. In NHEJ, the more common pathway, repair disrupts the target sequence by generation of small insertions or deletions, which collectively are called “indels”, with the result often a frameshift / loss of function mutation [23]. In contrast, HDR uses a template of donor DNA with a homologous sequence that serves as a matrix for the repair to take place at either cleaved end. HDR requires cells to be dividing and, although more controlled, is typically less efficient [24].

### Base editors, prime editors, and transposases

Correcting point mutations by inducing double strand DNA breaks is notoriously error prone due to the associated random insertions / deletions at the target locus as a cellular response to dsDNA breaks. ‘Base editing’ is a newer genome editing approach, enabling direct, irreversible conversion of one target DNA base into another, in a programmable and efficient manner [25]. Base editing is now a well-established technique, achieving high efficiency in dividing and non-dividing cells in vitro and in vivo. However, the method does have the potential to generate undesired off-target mutations when multiple target nucleotides exist within the base editing window [26]. Further CRISPR–Cas genome editing technologies include ‘prime editors’ which can introduce all 12 types of point mutations, in contrast to base editors which can only create single base substitutions for four transitions and 2 transversions. Prime editors can also achieve small insertions and small deletions in a precise and targeted manner, potentially addressing almost 90% of known disease-causing mutations [27]. Prime editing may be one of the most promising developments in genetic engineering since the Nobel prise-winning discovery of ‘genetic scissors’, (CRISPR-Cas9 system) [28]. and although it is currently less efficient than base editing, it appears to have higher ratios of correct editing to off target effects. Prime editing may therefore have advantages from a safety perspective and thus be more useful from a translational prospective as researchers try to bring new therapeutics from bench to bedside [29].

Emerging CRISPR-associated transposases (CASTs) allow recombination-independent multi kilobase DNA insertions at RNA programmed genomic locations, without double stranded DNA breaks [30]. The recent development of HELIX (a nicking homing endonuclease fusion to TnsB) has shown improved integration product purity and genome-wide specificity [31]. However, more research is required to develop methods to enhance integration efficiency before this technique becomes plausible clinically.

Each editing strategy has strengths and weaknesses, and selection of the optimal CRISPR–Cas tool hinges on various factors, including the targeted cell type, the cellular environment, and method of delivery [32].

### Cell reprogramming

Cell reprogramming represents an additional approach in the field of genetic engineering. The fate of a somatic cell was historically believed to be tightly controlled and progressively restricted with differentiation over time. The demonstration of the ability to alter the fate or identity of differentiated cells has contributed to a new age in regenerative medicine [33, 34]. Reprogramming of a cell can occur via the isolation of somatic cells (ex-vivo), their in-vitro reprogramming and subsequent transplantation back to source (induced pluripotent stem cells) or, perhaps more attractively, via direct reprogramming of cells in-vivo, also known as transdifferentiation [35]. As direct reprogramming takes place within the native cellular milieu, cells are exposed to tissue specific mechanical and biochemical signals, potentially leading to a more mature and effective cell conversion [36]. This approach is challenging, not least because the extracellular environment in injury or disease is often far from ideal, and there is also the need for sufficient healthy cells to reprogramme without other adverse effects on tissue function. Molecular mechanisms that enable cell reprogramming include transcriptional factors, epigenetic modifications (e.g. histone methylation), and more recently, non-coding RNA’s and metabolic factors [35]. Direct cell reprogramming is facilitating exciting advances in the realm of neurological regeneration and has demonstrated successful vision restoration in mouse models of congenital blindness [37]. Through gene transfer of β-catenin, cell-cycle-reactivated Müller glia were reprogrammed to generate rod photoreceptors by subsequent gene transfer of transcription factors essential for rod cell fate specification and determination, leading to restoration of vision.

### Optogenetics

Optogenetics is a further strategy for gene therapy and may have particular value in conditions where there is an insufficient cell population for reprogramming. Optogenetics employs light to selectively manipulate molecular events within cells. Specifically, genetically encoded proteins undergo conformational changes in response to light, thereby influencing cell behaviour, for example by altering the membrane voltage potential of excitable cells. In 2010, optogenetics was deemed ‘Method of the Year’ across all fields of science and engineering in *Nature Methods* [38], was listed in ‘Breakthroughs of the Decade’ by *Science* [39], and by 2021 the first medical use of optogenetics partially restored vision in a blind patient [40]. An adeno-associated viral vector encoding ChrimsonR was injected into the eye of a 58-year-old patient suffering from Retinitis Pigmentosa for 40 years, combined with light stimulation via engineered goggles to activate optogenetically transduced retinal ganglion cells (RGCs). The patient perceived, located, counted, and touched different objects using the vector-treated eye alone while wearing the goggles [40].

### Delivery vectors

Gene manipulating tools are delivered to target tissues by vectors, which can be viral or non-viral. Desirable vector characteristics include long term expression of transgenes, high expressivity, large carrying capacity and minimal risk of mutagenicity or immunogenicity [41, 42]. Viral vectors utilise the natural ability of viruses to infect and introduce genetic material into target cells and include adenoviruses (AV), adeno-associated viruses (AAV) and lentiviruses. Non-viral vectors include engineered structures that may incorporate lipids, peptides, inorganic molecules, polymers, and hybrids of multiple different components.

A viral vector is characterised by three integral components: the protein capsid and/or envelope that encases the genetic payload, determining the vector’s tissue or cell tropism and antigen recognition; the transgene of interest, which, when expressed in cells, imparts a desired effect; and a regulatory cassette, a composite of enhancer/promoter/auxiliary elements that governs stable or transient somatic expression of the transgene, either as an episome or a chromosomal integrant [41]. The most common viral vectors used in human studies to date have been AV and, increasingly, AAV.

AV vectors demonstrate a high transduction efficiency both in quiescent and dividing cells, a large packaging capacity, epichromosomal persistence in the host cell, and broad tropism for different tissue targets [43]. However, challenges of AV vectors include the prevalence of pre-existing immunity against human AV serotypes, and safety concerns related to immunogenicity and cellular toxicity [44]. For therapies not affected by immune response, or those aiming to kill transduced cells as in cancer therapy, AV vectors have an important role. It was a recombinant Ad-p53 that was the first commercialised gene therapy for cancer [45], and there has been a renewed interest in AV vectors despite initial setbacks, with their use accounting for 50% of global gene therapy trials to date, largely in vaccines and cancer therapies.

Adeno-associated viruses were discovered in 1965 as a contaminant of AV preparations [46]. Thirty years later, an AAV vector was used to deliver the cystic fibrosis transmembrane regulator (CFTR) gene packaged with the AAV2 capsid (rAAV2-CFTR), into a patient with cystic fibrosis [47]. There are now over a thousand known AAV variants. Compared to AV vectors, AAV vectors are less immunogenic, resulting in less vector related toxicity and undesired effects. AAVs are currently the most common vector in ocular gene therapy [48]. The existence of multiple serotypes confers increased flexibility to AAV vectors. These serotypes vary in their capsid components, leading to differences in transduction efficiency, immunogenicity, and cellular tropism [49]. AAV serotype 2 is commonly used when introducing transgenes into RGCs via intravitreal injection but has a limited carrying capacity for genetic cargo. Luxturna (voretigene neparvovec-rzyl) is an AAV2-based vector that delivers the retinoid isomerohydrolase RPE65 [6], the mutated gene in Leber’s congenital amaurosis, and was the first gene therapy treatment approved by the U.S. Food and Drug Administration (FDA) following improved functional vision in a previously untreatable retinal dystrophy in human clinical trials. Although vectors are of critical importance in carrying the genetic message for gene therapy into the target cell, promotors, enhancing elements and internal terminal repeats are also essential for effective transduction. Recent research has highlighted the influence of various promotors on the effectiveness, strength, and cell-selectivity of transgene expression [50], and has helped optimise AAV-mediated gene transduction particularly in increasing gene cargo size, a known limitation of AAV vectors [51].

### Delivery techniques

Administration of vectors can be in vivo or ex vivo. In vivo gene therapy denotes the direct delivery of a gene packaged into a vector, with the transgene delivered directly to target cells within the patient. Ex-vivo denotes the practice of harvesting target cells from the patient, applying the gene and vector, and reintroducing these cells to the patient. The preferred route of gene therapy delivery depends on the location of target cells. The most common routes include intravitreal and subretinal injections, but suprachoroidal delivery is emerging as a technique that may reduce the surgical challenges of subretinal delivery. Each of these approaches presents distinct advantages and disadvantages, including ease of access (outpatient setting for intravitreal vs operating theatre setting for subretinal), risk of iatrogenic damage (more challenging in subretinal applications) and duration of effect without eliciting immune reactions. Furthermore, the presence of the internal limiting membrane (ILM) can act as a barrier to viral vectors entering the neurosensory retina following intravitreal delivery, although emerging AAV subtypes may possibly overcome this hurdle to some extent [52]. An attractive aspect of subretinal delivery is access to a relatively immune privileged location without disruption of the blood brain barrier. This results in lower risks of eliciting unwanted immune responses. However, access typically requires a transvitreal approach in an operating theatre with a pars plana vitrectomy which is technically demanding and time-consuming. Transscleral access to the subretinal space via a suprachoroidal approach is possible using microneedles, and a number of recent studies have used this route [53, 54]. The delivery of agents to the suprachoroidal space is also an option, although rapid egress via the adjacent highly vascular choroid is potentially problematic. Using formulations with larger particle size such as steroid emulsions, viral particles, or biodegradable nanoparticles may help overcome this challenge [55].

Of note, applications of gene therapy to the cornea and anterior chamber also exist, with promising treatment strategies for Herpetic stromal keratitis (HSK) and Fuchs endothelial corneal dystrophy (FECD) [7]. HSK is a potentially blinding infectious condition which is often recurrent and difficult to treat with topical or systemic applications of antiviral treatments. Recently, intrastromal injection of HSV-1-erasing lentiviral particles (HELP) efficiently blocked HSV-1 replication and recurrence in three different animal models [56]. Recent research into gene therapy for FECD has demonstrated the potential of CRISPR‒Cas9-mediated to target the Col8a2 mutation in the early-onset FECD mouse model [57]. A single anterior-chamber injection of an adenovirus encoding SpCas9 and sgRNA led to significant improvements in corneal endothelial cell density and a reduction in the formation of guttae-like structures compared to untreated eyes. Further topical or intracameral applications of treatments for gene therapy candidates for glaucoma exist and will be discussed below.

## Conditions amenable to gene therapy

Gene therapy offers new hope to patients with previously untreatable blinding conditions. Perhaps the most obvious candidates for gene therapy in the eye are inherited retinal diseases which have been trailblazers for translating gene therapy from bench to bedside. However, more prevalent conditions without a single gene defect such as glaucoma, age-related macular degeneration, and diabetic retinopathy are subject to exciting ongoing research. With a growing and ageing population, there is increasing demand for new therapies for age-related conditions that cause vision loss and blindness. Whilst Luxturna is the only FDA approved gene therapy treatment at present, there is much progress in both pre-clinical and clinical trials. Table 1 shows a list of clinical trials (clinicaltrials.gov) with their associated conditions, Intervention (drug/vector), NCT number, phase, and trial sponsor.

**Table 1:** Current ocular diseases being examined for use of gene therapy.

	Conditions	Intervention (Drug / Vector)	NCT Number	Phase	Sponsor
**Inherited Retinal Diseases**	**Achromatopsia**	*AAV - CNGB3 or AAV - CNGA3*	NCT03278873	1/2	MeiraGTx UK II Ltd
		*AAV - CNGB3*	NCT03001310	1/2	MeiraGTx UK II Ltd
		*AAV- CNGA3*	NCT03758404	1/2	MeiraGTx UK II Ltd
		*AGTC-402*	NCT02935517	1/2	Applied Genetic Technologies Corp
		*rAAV.hCNGA3*	NCT02610582	1/2	STZ eyetrial
		*rAAV2tYF-PR1.7-hCNGB3 (AGTC-401)*	NCT02599922	1/2	Applied Genetic Technologies Corp
	**Bietti’s Crystalline Dystrophy**	*VGR-R01*	NCT05399069	1	Beijing Tongren Hospital
		*rAAV2/8-hCYP4V2*	NCT04722107	1	Beijing Tongren Hospital
	**Choroideremia**	*4D-110*	NCT04483440	1	4D Molecular Therapeutics
		*AAV-mediated REP1 gene replacement*	NCT02407678	2	University of Oxford
		*AAV2-REP1 (10e11 vg)*	NCT02553135	2	Byron Lam
		*BIIB111*	NCT03507686	2	Biogen
		*BIIB111*	NCT03496012	3	Biogen
		*rAAV2.REP1*	NCT02671539	2	STZ eyetrial
		*rAAV2.REP1*	NCT01461213	1/2	University of Oxford
		*rAAV2.REP1*	NCT02077361	1/2	University of Alberta
		*AAV2-hCHM*	NCT02341807	1/2	Spark Therapeutics, Inc.
	**Leber Congenital Amaurosis**	*rAAV2-CBSB-hRPE65*	NCT00481546	1	University of Pennsylvania
		*AAV RPE65*	NCT02781480	1/2	MeiraGTx UK II Ltd
		*AAV2-hRPE65v2,voretigene neparvovec-rzyl*	NCT00999609	3	Spark Therapeutics, Inc.
		*HG004*	NCT06088992	1	Xinhua Hospital, Shanghai Jiao Tong University
		*rAAV2-CB-hRPE65*	NCT00749957	1/2	Applied Genetic Technologies Corp
		*rAAV2-hRPE65*	NCT00821340	1	Hadassah Medical Organisation
		*rAAV2/4.hRPE65*	NCT01496040	1/2	Nantes University Hospital
		*AAV2-hRPE65v2,voretigene neparvovec-rzyl*	NCT04516369	3	Novartis Pharmaceuticals
		*AAV2-hRPE65v2,voretigene neparvovec-rzyl*	NCT00516477	1	Spark Therapeutics, Inc.
		*AAV OPTIRPE65*	NCT02946879	N/A	MeiraGTx UK II Ltd
		*tgAAG76 (rAAV 2/2.hRPE65p.hRPE65)*	NCT00643747	1/2	University College, London
		*QR-110*	NCT03140969	1/2	ProQR Therapeutics
		*LX101*	NCT06024057	N/A	Shanghai General Hospital
		*QR-110*	NCT03913130	1/2	ProQR Therapeutics
		*sepofarsen*	NCT03913143	2/3	ProQR Therapeutics
		*sepofarsen*	NCT04855045	2/3	ProQR Therapeutics
		*EDIT-101*	NCT03872479	1/2	Editas Medicine, Inc.
		*HG004*	NCT05906953	1/2	HuidaGene Therapeutics Co., Ltd.
		*AAV8.hLCA5*	NCT05616793	1/2	Opus Genetics, Inc
		*ATSN-101*	NCT03920007	1/2	Atsena Therapeutics Inc.
		*OCU400*	NCT05203939	1/2	Ocugen
	**Retinitis Pigmentosa**	*RST-001*	NCT02556736	1/2	AbbVie
		*GS030-DP / GS030-MD*	NCT03326336	1/2	GenSight Biologics
		*AAV2/5-hPDE6B*	NCT03328130	1/2	eyeDNA Therapeutics
		*BS01*	NCT04278131	1/2	Bionic Sight LLC
		*CPK850*	NCT03374657	1/2	Novartis Pharmaceuticals
		*MCO-010*	NCT04945772	2	Nanoscope Therapeutics Inc.
		*rAAV.hPDE6A*	NCT04611503	1/2	STZ eyetrial
		*rAAV2-VMD2-hMERTK*	NCT01482195	1	King Khaled Eye Specialist Hospital
		*SPVN06*	NCT05748873	1/2	SparingVision
		*VG901*	NCT06291935	1	ViGeneron GmbH
		*ZM-02-L*	NCT06292650	1	Zhongmou Therapeutics
		*vMCO-I*	NCT05921162	N/A	Nanoscope Therapeutics Inc.
		*vMCO-I*	NCT04919473	1/2	Nanoscope Therapeutics Inc.
		*MCO-010*	NCT06162585	N/A	Nanoscope Therapeutics Inc.
		*OCU400*	NCT05203939	1/2	Ocugen
		*QR-1123*	NCT04123626	1/2	ProQR Therapeutics
		*IVB102*	NCT06289452	1	InnoVec Biotherapeutics Inc.
		*RS1 AAV Vector*	NCT02317887	1/2	National Eye Institute (NEI)
	**IRD due to RPE65 Mutations**	*LX101*	NCT06196827	1/2	Innostellar Biotherapeutics Co.,Ltd
		*AAV2-hRPE65v2*	NCT03602820	N/A	Spark Therapeutics, Inc.
	**Stargardt Disease**	*MCO-010*	NCT06048185	N/A	Nanoscope Therapeutics Inc.
		*vMCO-010*	NCT05417126	2	Nanoscope Therapeutics Inc.
		*JWK006*	NCT06300476	1/2	West China Hospital
		*OCU410ST*	NCT05956626	1/2	Ocugen
		*SAR422459*	NCT01367444	1/2	Sanofi
		*SAR422459*	NCT01736592	1/2	Sanofi
	**Usher Syndrome**	*SAR421869*	NCT01505062	1/2	Sanofi
		*RNA antisense oligonucleotide for intravitreal injection*	NCT05085964	2	ProQR Therapeutics
		*QR-421a*	NCT03780257	1/2	ProQR Therapeutics
		*QR-421a*	NCT05176717	2/3	ProQR Therapeutics
		*Ultevursen*	NCT05158296	2/3	ProQR Therapeutics
	**X-Linked Retinitis Pigmentosa**	*4D-125*	NCT04517149	1/2	4D Molecular Therapeutics
		*AAV2/5-RPGR*	NCT03252847	1/2	MeiraGTx UK II Ltd
		*AAV5-hRKp.RPGR*	NCT04312672	N/A	Janssen Research & Development, LLC
		*AAV5-hRKp.RPGR*	NCT04671433	3	Janssen Research & Development, LLC
		*AAV5-hRKp.RPGR*	NCT04794101	3	Janssen Research & Development, LLC
		*AGTC-501*	NCT06275620	2	Beacon Therapeutics
		*BIIB112*	NCT03116113	1/2	Biogen
		*FT-002*	NCT05874310	1	Frontera Therapeutics
		*rAAV2tYF-GRK1-hRPGRco*	NCT04850118	2/3	Applied Genetic Technologies Corp
		*rAAV2tYF-GRK1-RPGR*	NCT03316560	1/2	Beacon Therapeutics
	**X-linked Retinoschisis**	*ZM-01-L / ZM-01-H*	NCT06066008	1	Zhongmou Therapeutics
		*ATSN-201*	NCT05878860	1/2	Atsena Therapeutics Inc.
		*LX103*	NCT05814952	N/A	Shanghai General Hospital
		*rAAV2tYF-CB-hRS1*	NCT02416622	1/2	Applied Genetic Technologies Corp
	**Choroideremia** | **X-Linked Retinitis Pigmentosa**	*BIIB111 / BIIB112*	NCT03584165	3	NightstaRx Ltd, a Biogen Company
**Age-related Macular Degeneration**	**Geographic Atrophy**	*GT005*	NCT05481827	2	Gyroscope Therapeutics Limited
		*AAV5-hRORA*	NCT06018558	1/2	Ocugen
	**Dry**	*AAVCAGsCD59*	NCT03144999	1	Janssen Research & Development, LLC
	**Neovascular (Wet)**	*KH631*	NCT05657301	1	Chengdu Origen Biotechnology Co., Ltd.
		*KH631*	NCT05672121	1/2	Chengdu Origen Biotechnology Co., Ltd.
		*NG101 AAV*	NCT05984927	1/2	Neuracle Genetics, Inc
		*RGX-314 / Ranibizumab*	NCT04704921	2/3	AbbVie
		*RGX-314 / Aflibercept*	NCT05407636	3	AbbVie
		*AAV2-sFLT01*	NCT01024998	1	Genzyme, a Sanofi Company
		*rAAV.sFlt-1*	NCT01494805	1/2	Lions Eye Institute, Perth, Western Australia
		*4D-150 / Aflibercept*	NCT05197270	1/2	4D Molecular Therapeutics
		*EXG102-031*	NCT05903794	1	Exegenesis Bio
		*ADVM-022*	NCT05536973	2	Adverum Biotechnologies, Inc.
		*FT-003*	NCT05611424	1	Frontera Therapeutics
		*LX102*	NCT06198413	1	Innostellar Biotherapeutics Co.,Ltd
		*LX102*	NCT06196840	2	Innostellar Biotherapeutics Co.,Ltd
		*RRG001*	NCT06141460	1/2	Shanghai Refreshgene Technology Co., Ltd.
		*SKG0106*	NCT06213038	1	Youxin Chen
		*SKG0106*	NCT05986864	1/2	Skyline Therapeutics (US) Inc.
		*RGX-314 Dose / Ranibizumab*	NCT04514653	2	AbbVie
		*HG202*	NCT06031727	1	HuidaGene Therapeutics Co., Ltd.
		*RGX-314*	NCT03066258	1/2	REGENXBIO Inc.
		*RGX-314*	NCT03999801	2	AbbVie
		*RGX-314*	NCT04832724	2	AbbVie
		*LX109*	NCT06022744	N/A	Shanghai General Hospital
		*AAVCAGsCD59*	NCT03585556	1	Janssen Research & Development, LLC
		*ADVM-022*	NCT04645212	N/A	Adverum Biotechnologies, Inc.
		*BD311*	NCT05099094	1	Shanghai BDgene Co., Ltd.
		*ADVM-022*	NCT03748784	1	Adverum Biotechnologies, Inc.
**Diabetic Retinopathy (DR)**	**Diabetic Macular Oedema**	*ADVM-022*	NCT05607810	N/A	Adverum Biotechnologies, Inc.
		*FT-003*	NCT05916391	1	Frontera Therapeutics
		*SKG0106*	NCT06237777	1	Wang Min
		*6E11 vg/eye of ADVM-022 / Aflibercept*	NCT04418427	2	Adverum Biotechnologies, Inc.
		*OCU200*	NCT05802329	1	Ocugen
		*4D-150 / Aflibercept*	NCT05930561	2	4D Molecular Therapeutics
	**DR without centre involving Macular Oedema**	*RGX-314*	NCT04567550	2	AbbVie
**Ocular oncology**	**Metastatic Uveal Melanoma**	*ADV/HSV-tk / Valacyclovir / SBRT / nivolumab*	NCT02831933	2	Eric Bernicker, MD
	**Retinoblastoma, Recurrent**	*VCN-01*	NCT03284268	1	Fundaci√≥ Sant Joan de D√©u
	**Eye; Melanoma**	*TCR transduced T-cells*	NCT02654821	1/2	The Netherlands Cancer Institute
**Leber Hereditary Optic Neuropathy (LHON)**		*rAAV2-ND4*	NCT03428178	N/A	Bin Li
		*GS010*	NCT02064569	1/2	GenSight Biologics
		*GS010*	NCT03406104	3	GenSight Biologics
		*GS010*	NCT03293524	3	GenSight Biologics
		*rAAV2-ND4*	NCT01267422	N/A	Bin Li
		*rAAV2-ND4*	NCT03153293	2/3	Huazhong University of Science and Technology
		*NFS-02*	NCT05820152	1/2	Neurophth Therapeutics Inc
		*NR082*	NCT05293626	1/2	Neurophth Therapeutics Inc
		*scAAV2-P1ND4v2*	NCT02161380	1	Byron Lam
		*NR082*	NCT04912843	2/3	Wuhan Neurophth Biotechnology Limited Company
**Neuronal Ceroid Lipofuscinosis**		*NGN-101*	NCT05228145	1/2	Neurogene Inc.
		*RGX-381*	NCT05791864	1/2	REGENXBIO Inc.
**Viral Keratitis**		*BD111*	NCT04560790	N/A	Shanghai BDgene Co., Ltd.

*NCT* National Clinical Trial.

### Inherited retinal diseases

Inherited retinal diseases (IRD) are the most common cause of legal blindness in the working age population, with a prevalence of approximately 1 in 3000, and they present a significant socioeconomic burden [58–60]. IRDs are a complex and varied group of disorders which together lead to irreversible vision loss through progressive loss of photoreceptors and/or retinal pigment epithelium (RPE) cells. As a phenotypically diverse group, they are unsurprisingly genetically heterogenous, comprising over 300 different single gene defects. IRDs vary according to their age of onset, with some presenting at birth, others in early adolescence, or adulthood. IRDs also differ in the region of retina more affected, whether they are stationary or progressive, the inheritance pattern, and the extent of extraocular involvement as part of a wider syndrome affecting other organs. Patients with IRDs were previously offered genetic testing for prognosis alone, and treatment was often limited to low vision rehabilitation. However, advances in gene therapy, stem cell therapy and retinal prostheses herald a new age of hope [61]. In terms of emerging therapies for IRD, the most appropriate strategy depends on the stage of the disease. In early rod/cone disease where cell populations are intact, gene specific therapies and DNA editing may show promise. As photoreceptors and retinal pigment epithelium are progressively lost, RNA therapies, translational read-through therapies, stem cell therapies and optogenetics may play an increasingly relevant role.

### Leber congenital amaurosis (LCA)

LCA is the most common form of inherited blindness in children. It is a progressive, recessively inherited, rod-cone dystrophy characterised clinically by severe congenital or early infancy vision loss, nystagmus, amaurotic pupils, and severely abnormal full-field electroretinography [62]. Causative genes can be identified in around 80% of cases at present, reflecting our current incomplete understanding of disease causing variants, although more than 20 genes have been identified to date that are associated with the disease [63]. Luxturna (voretigene neparvovec-rzyl), an AAV2-based therapy, was a landmark treatment option for patients with biallelic loss of function mutations in RPE65, and was the first gene therapy treatment approved by the U.S. Food and Drug Administration (FDA) following demonstration of improved functional vision in previously untreatable LCA [6]. An alternative strategy to treat RPE65 related disease has involved subretinal injection of adeno-associated virus carrying CRISPR-Cas9 and donor DNA in a mouse model. However, although this technique achieved improved a and b waves on electroretinogram 7 months after injection, the researchers found poor correction efficiencies and an unacceptable rate of indel formation [64].

However, more recent use of base editing technology has shown promising results. In adult mice, a subretinal injection of a lentivirus expressing an adenine base editor and a single-guide RNA targeting a de novo nonsense mutation in the Rpe65 gene corrected the pathogenic mutation with up to 29% efficiency, less than 0.5% indel formation and minimal off-target mutations [65]. Treated mice displayed restored RPE65 expression and retinoid isomerase activity, with near-normal levels of retinal and visual function. The high level of precision achieved with prime editing has also recently been employed in 2 mouse models of LCA, with dual AAV delivery to the subretinal space [66, 67] correcting pathogenic mutations with up to 16% efficiency, with no detectable off-target edits, restoring RPE65 expression, rescuing retinal and visual function, and preserving photoceptors. These results provide motivation for further work to evaluate base editing and prime editing for therapeutic applications in LCA.

### Retinitis pigmentosa (RP)

RP is the most common IRD, with a prevalence of 1 in 4000, over 80 genes with disease causing variants, and varied patterns of inheritance [68]. Rods are initially affected, with their dysfunction manifesting as night blindness, and subsequent cone dysfunction leading to central vision loss, with photoreceptor dysfunction producing a markedly diminished electroretinogram.

Gene therapy for RP can employ several different techniques based on inheritance pattern. For example, gene replacement strategies can compensate for biallelic inheritance of recessive loss of function mutations, but this strategy would be ineffective in autosomal dominant RP, where a monoallelic gain of function mutation exists [69]. For autosomal dominant RP, where a single mutant allele causes dysfunctional protein, the CRISPR/Cas9 mediated NHEJ repair has been shown to be effective in a rat model, inducing a double stranded break and causing mutations that disrupt the mutant allele [70]. A single subretinal injection of an sgRNA specific to the mutant allele along with the SpCas9 plasmid disrupted the mutant allele, preserved the wild type functional allele, prevented retinal degeneration and improved visual function.

Prime editing has recently been utilised to prevent vision loss caused by RP-associated gene mutations in Pde6b mouse models [71]. Qin and colleagues developed a genome-editing tool characterised by the versatility of prime editors (PEs) and unconstrained PAM requirement of a SpCas9 variant (SpRY), referred to as PESpRY. The diseased retinas of Pde6b-associated RP mice were transduced via a dual AAV system packaging PESpRY. Progressive cell loss was reversed, leading to substantial rescue of photoreceptors and production of functional PDE6β. The treated mice exhibited significantly improved ERG responses and displayed good performance in both passive and active avoidance tests. Moreover, they had an apparent improvement in visual stimuli-driven optomotor responses and efficiently completed visually guided water-maze tasks. The high rate of mutation correction with low indel rates achievable with prime editing justify its place at the forefront of emerging gene editing strategies for IRD.

### Stargardt disease (STGD)

STGD, the most prevalent inherited macular dystrophy, is caused by mutations in the adenosine triphosphate binding cassette transporter subfamily A member 4 (ABCA4) gene and is inherited in an autosomal recessive pattern [72]. Clinical characteristics vary widely both in age of onset, rate of progression and severity of symptoms. Over 1500 pathogenic variants of the ABCA4 gene have been identified, of which most are missense and nonsense variants [73]. Gene therapy for STGD aims to introduce a functional ABCA4 gene, capable of producing an adequate amount of the standard, active transporter protein in photoreceptor cells, thereby preventing disease progression. The ABCA4 gene is large (7 kb) and thus the limited carrying capacity of AAV (4.7 kb) requires a dual vector strategy whereby large genes are split into two halves and packaged in two separate AAV vectors [74]. Human induced pluripotent stem cells (hiPSCs) constitute a readily available source of patient-derived cells. The combination of hiPSCs and CRISPR-Cas9 technology enables in vitro gene editing in patient-derived cells to correct their specific mutations, allowing their differentiation into retina cells for autologous transplantation. Siles et al. have demonstrated efficient gene editing in two STGD related ABCA4 pathogenic variants, through single-stranded oligodeoxynucleotides (ssODNs) mediated repair, in human induced pluripotent stem cells from two unrelated patients affected with Stargardt disease [75]. Gene editing was achieved with no detectable off-target genomic alterations, demonstrating efficient ABCA4 gene correction without deleterious effects.

### Age related macular degeneration and diabetic retinopathy

Many of the gene therapy strategies discussed so far involve replacement of faulty genes. However in conditions such as AMD and DR, it has been suggested that gene therapy with anti-vascular endothelial growth factor (anti-VEGF) could achieve lifelong treatment with a single intravitreal injection, avoiding the need for repeated injections [76]. This would be of huge value given the growing prevalence of these chronic conditions and the inability for many resource poor healthcare systems to deal with growing treatment burdens. Indeed, there is often a disparity between real world and clinical trial results for current anti-VEGF treatments which may reflect the difficulties for patients and health systems of delivering such frequent, expensive, and invasive therapies [77–79].

Emerging treatment options for long term modulation of VEGF include RGX-314 (Regenxbio), and ADVM-022 (Adverum Biotechnologies). RGX-314 uses AAV8 to deliver genetic code expressing a monoclonal antibody fragment similar to ranibizumab, an established anti VEGF treatment which binds VEGF-A, supressing choroidal neovascularisation. The subretinal delivery is being examined in the ATMOSPHERE trial [80], whilst AAVIATE [81] and ALTITUDE [82] for AMD and diabetic retinopathy respectively, explore RGX-314 delivered via the suprachoroidal route [76]. So far results indicate it is efficacious, durable and safe [83]. Notably, in the AAVVIATE trial, following increased dosing across 5 cohorts, the amount of anti-VEGF produced within the eye increased in a dose dependent fashion, with the highest dose cohort demonstrating a 85% reduction in treatment burden for year 1, and a 79% reduction in treatment burden at year 2 [83].

ADVM-022 aims to treat nAMD through a single intravitreal injection utilising the AAV.7m8 capsid to deliver a codon-optimised cDNA expressing an aflibercept-like protein. It has shown long term safety and durable efficacy in non-human primate models [52] and results from the ongoing OPTIC trial show promising results for use in humans [84]. Indeed, over 80% of patients with nAMD treated with a single injection of ADVM-022 in OPTIC did not require any supplemental anti-VEGF injections up to 92 weeks follow-up [84]. Importantly, any secondary inflammation seemed to occur in the anterior segment and was responsive to topical steroids, in comparison to the more severe inflammatory responses and hypotony observed within the INFINITY trial of ADVM-022 for diabetic macular oedema which led to early termination of the trial.

Dry AMD is responsible for most of the visual impairment from AMD, and although it accounts for up to 90% of cases of AMD, there are currently few treatment options [85]. Efforts to target the dysregulated complement system, believed to be a key feature of dry AMD, may give rise to hope for novel treatments. One example is GT-005, whereby an AAV2 vector delivers a plasmid construct expressing normal Complement Factor I (CFI) protein, a natural inhibitor of the complement system [86]. Whilst phase 2 trials evaluating the safety and efficacy of GT-005, such as FOCUS [87] and EXPLORE [88] are ongoing, HORIZON [89] recently terminated following interim analysis demonstrating futility.

Another gene therapy treatment under investigation for advanced dry AMD employs an AAV2 vector expressing sCD59, administered intravitreally 7 days after a single intravitreal injection of anti-VEGF treatment. It aims to upregulate CD59 expression in RPE cells, protecting against a dysregulated complement cascade and membrane attack complex formation. In a phase 1, open-label, multicentre, dose-escalation, safety and tolerability study a single intravitreal injection of JNJ-81201887 (JNJ-1887), (formerly referred to as AAVCAGsCD59), in patients with advanced non-exudative AMD with geographic atrophy (GA), showed a continual decline in lesion growth over six-month increments [90]. For treated eyes in the high dose cohort, GA lesion growth rate showed continued decline through 24 months, with a reduction in mean square root lesion growth from 0.211 mm at months 0-6 to 0.056 mm at months 18-24 [90]. All 17 patients met safety endpoints at 2 years without steroid prophylaxis [91]. The Phase 2b PARASOL clinical trial [92] is currently actively enroling patients. JNJ-1887 has been granted Fast Track designation by the U.S. Food and Drug Administration (FDA) and Advanced Therapy Medicinal Product (ATMP) designation by the European Medicines Agency (EMA). The long-term safety and efficacy of these treatment modalities will be followed with much interest as they continue to be studied.

### Glaucoma

Glaucoma is the leading cause of irreversible blindness worldwide [93]. Damage to RGCs results in progressive visual field loss and ultimate blindness. The aetiology of glaucoma is incompletely understood, although intraocular pressure remains the main modifiable risk factor. Genome wide association studies have implicated hundreds of gene loci in predisposing an individual to developing glaucoma [94].

Regardless of the subtype of glaucoma, all current treatment options rely on medical or surgical IOP lowering, either by reducing aqueous production or increasing outflow (trabecular or uveoscleral). Many patients are treated with daily applications of topical medications which may be expensive and are often associated with side effects, intolerance and poor adherence.

### IOP modulation via reduced aqueous production

Topical prostaglandin analogues are very effective at reducing eye pressure. The observation that glaucomatous individuals have lower levels of cyclooxygenase-2 (COX2) in the nonpigmented secretory epithelium of the ciliary body [95], an essential, rate limiting enzyme for prostaglandin synthesis, has led to the suggestion that gene therapy to introduce a transgene for the prostaglandin F2α receptor and COX2 could potentially reduce the burden of daily prostaglandin drops and circumvent issues of poor tolerance and adherence. IOP lowering has been effectively demonstrated following intracameral delivery of recombinant adeno-associated viral vector-mediated gene therapy (rAAV2/2[MAX].CCPP) which leads to de novo biosynthesis of prostaglandin F2alpha within the anterior chamber [96]. Interestingly, a dose dependent IOP lowering effect was seen in normotensive rats over a 12 month period which could be temporarily halted through off-type riboswitch activation, reverting intraocular pressure to normal. Further attempts to target genes implicated in regulating aqueous production have looked at Aquaporin 1, a water channel protein expressed in the ciliary body epithelium. In mouse models, adeno-associated virus ShH10 serotype has been used to deliver a CRISPR-Cas9 system disrupting Aquaporin 1 in the ciliary body epithelium, resulting in lowered IOP, and in experimental models of corticosteroid and microbead-induced ocular hypertension, lowering IOP resulted in less RGC loss [97]. Furthermore, ShH10 could transduce human ciliary body from post-mortem donor eyes in ex vivo culture with indel formation detectable in the Aquaporin 1 locus [97]. Gene silencing techniques have also demonstrated success in IOP lowering in animal models. Blocking beta2 adrenergic receptor (ADRB2) reduces IOP by decreasing production of aqueous at the ciliary body, and thus the ADRB2 silencing siRNA, SYL040012, has been shown to successfully reduce ADRB2 expression and lower IOP in normotensive and elevated IOP rabbit models [98]. An important safety consideration in gene silencing techniques, SYL040012 was actively taken up by cells of the ciliary body but not by cells of systemic organs such as the lungs, where inhibition of ADRB2 could cause undesirable side effects.

### IOP lowering via increased aqueous outflow

Gene therapy IOP modulation through increased aqueous outflow has been effective in animal models via targeting of matrix metalloproteinases (MMP). MMP are key regulators for remodelling extracellular matrices in the juxtacanalicular connective tissue region of the trabecular meshwork, and their dysregulation is implicated in POAG [99]. Reduced levels of MMP-3 activity have been demonstrated in the aqueous humour of glaucomatous individuals [100]. Intracameral inoculation of AAV2/9 containing a CMV-driven MMP-3 gene (AAV-MMP-3) into wild type mice has shown efficient transduction of corneal endothelium, increased aqueous concentration and activity of MMP-3, leading to increased outflow facility, and decreased IOP via degradation and remodelling of core extracellular matrix components [100]. Similarly, a single intracameral injection of a glucocorticoid-inducible adenovirus vector carrying a human *MMP-1* gene (AdhGRE.MMP1) lowered IOP by 70% in a steroid-induced ocular hypertension model in sheep [101].

Mutations in the myocilin (MYOC) gene have long been implicated in glaucoma, contributing to 4% of POAG cases and accounting for over 30% of cases involving adult-onset juvenile glaucoma [102]. MYOC gain of function mutations lead to protein misfolding and endoplasmic reticulum (ER) stress in the trabecular meshwork (TM), thus affecting aqueous outflow / IOP, and so reduction of mutant MYOC is an attractive therapeutic target. As there are numerous different pathogenic *MYOC* mutations [103], gene editing to introduce an early frame shift mutation at the start codon of human MYOC gene should result in termination of the protein and be equally effective treatment for various MYOC mutations. Indeed, using CRISPR-Cas9 via guide RNA (Ad5-crMYOC) it is possible to disrupt the MYOC gene and its aberrant function in human and mouse TM cells, as well as in human ex vivo perfusion-cultured eyes [104]. In mutant *MYOC* ocular hypertensive mice, intravitreal injection of (Ad5-crMYOC) prevented IOP elevation, ER stress, and subsequent glaucomatous damage, and furthermore lowered IOP when already raised for 9 months prior to treatment [104]. Improvements in RGC function, as measured by pattern electroretinography were also noted.

Another gene associated with POAG is transforming growth factor-beta 2 (TGFβ2), which is much more abundant in the aqueous [105] and optic nerve head [106] of glaucomatous patients. Recently, a CRISPR interference system has been utilised to selectively deacetylate histones in the TGFβ2 gene promoter, reducing TGFβ2 expression in human TM cells and ameliorating ocular hypertension in a TGFβ2-overexpressing mouse model [107].

### IOP independent strategies – neuroprotection and neuroregeneration

IOP independent strategies such as neuroprotection are attractive, as for many patients conventional IOP lowering treatments are ineffective in halting progression, or are limited by side effects or adherence issues. Gene therapy is one potential way to achieve a long-term therapeutic neuroprotective effect as an adjunct to IOP lowering treatment. Activation of pro-survival cell-signalling pathways by modulation of brain-derived neurotrophic factor (BDNF) signalling has been suggested as a possible treatment for glaucoma[108, 109]. RGC protection after optic nerve injury has been achieved by BDNF supplementation, whether through injection of recombinant protein [110, 111] or through gene therapy approaches [112], but the duration of effect is adversely affected by BDNF receptor (TrkB) downregulation [113, 114]. Recently, intravitreal delivery of an adeno-associated virus (AAV) gene therapy (AAV2 TrkB-2A-mBDNF) has been shown to increase both BDNF production, and TrkB expression within the inner retina, improving long-term neuroprotective signalling [115]. Furthermore, the simultaneous overexpression of BDNF and TrkB by a single vector with one promoter, is more effective in stimulating anterograde axonal transport than either receptor administration or ligand administration alone [116]. This approach has demonstrated significant and sustained elevation of survival signalling pathways ERK and AKT within RGCs over 6 months, avoiding receptor downregulation in a mouse model of optic nerve crush (ONC) and a rat model of chronic IOP elevation. Furthermore, there were no adverse effects of the vector on retinal structure or electrophysiological function in young or aged animals. Moreover, treatment with AAV2 TrkB-2A-mBDNF remains effective if administered after the onset of pathology, suggesting a clinically relevant therapeutic window.

Further attempts to avoid the transient nature of ligand-dependent activation of neurotrophic factor signalling have also been made. Forcing membrane localisation of the intracellular domain of tropomyosin receptor kinase B (iTrkB), results in constitutive activation and induction of downstream signalling without the need for ligands such as BDNF [117]. Delivery of intraocular AAV-F-iTrkB, has been shown to lead to an iTrkB mediated neuroprotection in mouse models of glaucoma and stimulates robust axon regeneration after optic nerve injury. Additionally, in an optic tract transection model, in which the injury site was near the superior colliculus, iTrkB expression in the retina was also effective. Remarkably, regenerating axons were reported to reach targets in the brain, resulting in partial recovery of visual behaviour.

Adult central nervous system axons have intrinsically poor regenerative ability [118], and thus optic nerve damage in glaucoma is irreversible. However, much research is underway to try to enhance regeneration, for example through the axonal supply of growth molecules such as protrudin. Protrudin acts as a scaffolding molecule, bringing together multiple other molecules (growth factor receptors, motor proteins, late-endosomal proteins), organelles (ER, endosomes, lysosomes) and cellular components (lipids, membrane components) at the tip of growing axons – it helps ‘protrude’ the growth cone. Elevated Protrudin expression has been shown to enable robust central nervous system regeneration both in vitro in primary cortical neurons, and in vivo in the injured optic nerve [119]. Specifically, Petrova et al. generated three constructs for AAV delivery to mouse retina by intravitreal injection: AAV2-GFP, AAV2-ProtrudinGFP, and AAV2-phosphomimetic-Protrudin-GFP (phosphomimetic protrudin being the active form). They were introduced 2 weeks prior to ONC and transduced 40–45% of RGCs throughout the retina. Within 2 weeks of ONC, retinas expressing phosphomimetic Protrudin showed 52% RGC survival compared to 27% and 28% in those injected with GFP control or wild-type Protrudin respectively. In contrast, both wild-type and phosphomimetic Protrudin promoted axonal regeneration. Regenerating axons extended up to 2.75 mm in wild-type Protrudin-transduced animals, and as far as 3.5 mm in phosphomimetic Protrudin-transduced animals whilst the control group exhibited limited regeneration (0% >0.5 mm from the crush site). The number of regenerating axons was high for phosphomimetic Protrudin, in which over 630 axons were seen proximally, compared to 44 axons in the control group.

## Conclusions

Genetic engineering technology is providing revolutionary new approaches to the future treatment of eye disease, with publications and clinical trials in this field both increasing exponentially over the last 20 years [120]. Inherited ocular conditions resulting from single gene mutations and chronic polygenetic conditions are both amenable to gene therapy strategies, and previously untreatable conditions are now becoming treatable. The eye is at the very forefront in the application of genetic engineering strategies to treat disease, and further progress is awaited with great interest.

## References

[1] Hoermann A, Habtewold T, Selvaraj P, Del Corsano G, Capriotti P, Inghilterra MG, et al. Gene drive mosquitoes can aid malaria elimination by retarding Plasmodium sporogonic development. Sci Adv. 2022;8:eabo1733. 36129981 10.1126/sciadv.abo1733 PMC9491717

[2] Wang GH, Gamez S, Raban RR, Marshall JM, Alphey L, Li M, et al. Combating mosquito-borne diseases using genetic control technologies. Nat Commun. 2021;12:4388. 34282149 10.1038/s41467-021-24654-z PMC8290041

[3] Ali Q, Yu C, Hussain A, Ali M, Ahmar S, Sohail MA, et al. Genome engineering technology for durable disease resistance: recent progress and future outlooks for sustainable agriculture. Front Plant Sci. 2022;13:860281. 35371164 10.3389/fpls.2022.860281 PMC8968944

[4] Maynard LH, Humbert O, Peterson CW, Kiem HP. Genome editing in large animal models. Mol Ther. 2021;29:3140–52. 34601132 10.1016/j.ymthe.2021.09.026 PMC8571486

[5] Arabi F, Mansouri V, Ahmadbeigi N. Gene therapy clinical trials, where do we go? An overview. Biomed Pharmacother. 2022;153:113324. 35779421 10.1016/j.biopha.2022.113324

[6] Russell S, Bennett J, Wellman JA, Chung DC, Yu ZF, Tillman A, et al. Efficacy and safety of voretigene neparvovec (AAV2-hRPE65v2) in patients with RPE65-mediated inherited retinal dystrophy: a randomised, controlled, open-label, phase 3 trial. Lancet. 2017;390:849–60. 28712537 10.1016/S0140-6736(17)31868-8 PMC5726391

[7] Choi EH, Suh S, Sears AE, Holubowicz R, Kedhar SR, Browne AW, et al. Genome editing in the treatment of ocular diseases. Exp Mol Med. 2023;55:1678–90. 37524870 10.1038/s12276-023-01057-2 PMC10474087

[8] Ghoraba HH, Akhavanrezayat A, Karaca I, Yavari N, Lajevardi S, Hwang J, et al. Ocular gene therapy: a literature review with special focus on immune and inflammatory responses. Clin Ophthalmol. 2022;16:1753–71. 35685379 10.2147/OPTH.S364200 PMC9173725

[9] Kovacs KD, Ciulla TA, Kiss S. Advancements in ocular gene therapy delivery: vectors and subretinal, intravitreal, and suprachoroidal techniques. Expert Opin Biol Ther. 2022;22:1193–208. 36062410 10.1080/14712598.2022.2121646

[10] Ratican SE, Osborne A, Martin KR. Progress in gene therapy to prevent retinal ganglion cell loss in glaucoma and leber’s hereditary optic neuropathy. Neural Plast. 2018;2018:7108948. 29853847 10.1155/2018/7108948 PMC5954906

[11] Sulak R, Liu X, Smedowski A. The concept of gene therapy for glaucoma: the dream that has not come true yet. Neural Regen Res. 2024;19:92–9. 37488850 10.4103/1673-5374.375319 PMC10479832

[12] Lanigan TM, Kopera HC, Saunders TL. Principles of genetic engineering. Genes. 2020;11:291. 32164255 10.3390/genes11030291 PMC7140808

[13] Somia N, Verma IM. Gene therapy: trials and tribulations. Nat Rev Genet. 2000;1:91–9. 11253666 10.1038/35038533

[14] Woods NB, Bottero V, Schmidt M, von Kalle C, Verma IM. Gene therapy: therapeutic gene causing lymphoma. Nature. 2006;440:1123. 16641981 10.1038/4401123a

[15] Howe SJ, Mansour MR, Schwarzwaelder K, Bartholomae C, Hubank M, Kempski H, et al. Insertional mutagenesis combined with acquired somatic mutations causes leukemogenesis following gene therapy of SCID-X1 patients. J Clin Invest. 2008;118:3143–50. 18688286 10.1172/JCI35798 PMC2496964

[16] Dyer O. Researcher who edited babies’ genome retreats from view as criticism mounts. BMJ. 2018;363:k5113. 30504437 10.1136/bmj.k5113

[17] Lander ES, Baylis F, Zhang F, Charpentier E, Berg P, Bourgain C, et al. Adopt a moratorium on heritable genome editing. Nature. 2019;567:165–8. 30867611 10.1038/d41586-019-00726-5

[18] Urnov FD, Rebar EJ, Holmes MC, Zhang HS, Gregory PD. Genome editing with engineered zinc finger nucleases. Nat Rev Genet. 2010;11:636–46. 20717154 10.1038/nrg2842

[19] Joung JK, Sander JD. TALENs: a widely applicable technology for targeted genome editing. Nat Rev Mol Cell Biol. 2013;14:49–55. 23169466 10.1038/nrm3486 PMC3547402

[20] Sander JD, Joung JK. CRISPR-Cas systems for editing, regulating and targeting genomes. Nat Biotechnol. 2014;32:347–55. 24584096 10.1038/nbt.2842 PMC4022601

[21] Kocak DD, Josephs EA, Bhandarkar V, Adkar SS, Kwon JB, Gersbach CA. Increasing the specificity of CRISPR systems with engineered RNA secondary structures. Nat Biotechnol. 2019;37:657–66. 30988504 10.1038/s41587-019-0095-1 PMC6626619

[22] Ceccaldi R, Rondinelli B, D’Andrea AD. Repair pathway choices and consequences at the double-strand break. Trends Cell Biol. 2016;26:52–64. 26437586 10.1016/j.tcb.2015.07.009 PMC4862604

[23] Lieber MR. The mechanism of double-strand DNA break repair by the nonhomologous DNA end-joining pathway. Annu Rev Biochem. 2010;79:181–211. 20192759 10.1146/annurev.biochem.052308.093131 PMC3079308

[24] Heyer WD, Ehmsen KT, Liu J. Regulation of homologous recombination in eukaryotes. Annu Rev Genet. 2010;44:113–39. 20690856 10.1146/annurev-genet-051710-150955 PMC4114321

[25] Komor AC, Kim YB, Packer MS, Zuris JA, Liu DR. Programmable editing of a target base in genomic DNA without double-stranded DNA cleavage. Nature. 2016;533:420–4. 27096365 10.1038/nature17946 PMC4873371

[26] Rees HA, Liu DR. Base editing: precision chemistry on the genome and transcriptome of living cells. Nat Rev Genet. 2018;19:770–88. 30323312 10.1038/s41576-018-0059-1 PMC6535181

[27] Anzalone AV, Randolph PB, Davis JR, Sousa AA, Koblan LW, Levy JM, et al. Search-and-replace genome editing without double-strand breaks or donor DNA. Nature. 2019;576:149–57. 31634902 10.1038/s41586-019-1711-4 PMC6907074

[28] Jinek M, Chylinski K, Fonfara I, Hauer M, Doudna JA, Charpentier E. A programmable dual-RNA-guided DNA endonuclease in adaptive bacterial immunity. Science. 2012;337:816–21. 22745249 10.1126/science.1225829 PMC6286148

[29] Scholefield J, Harrison PT. Prime editing - an update on the field. Gene Ther. 2021;28:396–401. 34031549 10.1038/s41434-021-00263-9 PMC8376635

[30] Strecker J, Ladha A, Gardner Z, Schmid-Burgk JL, Makarova KS, Koonin EV, et al. RNA-guided DNA insertion with CRISPR-associated transposases. Science. 2019;365:48–53. 31171706 10.1126/science.aax9181 PMC6659118

[31] Tou CJ, Orr B, Kleinstiver BP. Precise cut-and-paste DNA insertion using engineered type V-K CRISPR-associated transposases. Nat Biotechnol. 2023;41:968–79. 36593413 10.1038/s41587-022-01574-x

[32] Anzalone AV, Koblan LW, Liu DR. Genome editing with CRISPR-Cas nucleases, base editors, transposases and prime editors. Nat Biotechnol. 2020;38:824–44. 32572269 10.1038/s41587-020-0561-9

[33] Takahashi K, Yamanaka S. Induction of pluripotent stem cells from mouse embryonic and adult fibroblast cultures by defined factors. Cell. 2006;126:663–76. 16904174 10.1016/j.cell.2006.07.024

[34] Davis RL, Weintraub H, Lassar AB. Expression of a single transfected cDNA converts fibroblasts to myoblasts. Cell. 1987;51:987–1000. 3690668 10.1016/0092-8674(87)90585-x

[35] Wang H, Yang Y, Liu J, Qian L. Direct cell reprogramming: approaches, mechanisms and progress. Nat Rev Mol Cell Biol. 2021;22:410–24. 33619373 10.1038/s41580-021-00335-z PMC8161510

[36] Gascon S, Masserdotti G, Russo GL, Gotz M. Direct neuronal reprogramming: achievements, hurdles, and new roads to success. Cell Stem Cell. 2017;21:18–34. 28686866 10.1016/j.stem.2017.06.011

[37] Yao K, Qiu S, Wang YV, Park SJH, Mohns EJ, Mehta B, et al. Restoration of vision after de novo genesis of rod photoreceptors in mammalian retinas. Nature. 2018;560:484–8. 30111842 10.1038/s41586-018-0425-3 PMC6107416

[38] Editorial. Method of the Year 2010. Nat Methods 8, 1 (2011). Nat Methods. 2010;8.

[39] News S. Insights of the decade. Stepping away from the trees for a look at the forest. Introduction. Science. 2010;330:1612–3. 21163985 10.1126/science.330.6011.1612

[40] Sahel JA, Boulanger-Scemama E, Pagot C, Arleo A, Galluppi F, Martel JN, et al. Partial recovery of visual function in a blind patient after optogenetic therapy. Nat Med. 2021;27:1223–9. 34031601 10.1038/s41591-021-01351-4

[41] Bulcha JT, Wang Y, Ma H, Tai PWL, Gao G. Viral vector platforms within the gene therapy landscape. Signal Transduct Target Ther. 2021;6:53. 33558455 10.1038/s41392-021-00487-6 PMC7868676

[42] Zu H, Gao D. Non-viral vectors in gene therapy: recent development, challenges, and prospects. AAPS J. 2021;23:78. 34076797 10.1208/s12248-021-00608-7 PMC8171234

[43] Lee CS, Bishop ES, Zhang R, Yu X, Farina EM, Yan S, et al. Adenovirus-mediated gene delivery: potential applications for gene and cell-based therapies in the new era of personalized medicine. Genes Dis. 2017;4:43–63. 28944281 10.1016/j.gendis.2017.04.001 PMC5609467

[44] Muruve DA. The innate immune response to adenovirus vectors. Hum Gene Ther. 2004;15:1157–66. 15684693 10.1089/hum.2004.15.1157

[45] Peng Z. Current status of gendicine in China: recombinant human Ad-p53 agent for treatment of cancers. Hum Gene Ther. 2005;16:1016–27. 16149900 10.1089/hum.2005.16.1016

[46] Atchison RW, Casto BC, Hammon WM. Adenovirus-associated defective virus particles. Science. 1965;149:754–6. 14325163 10.1126/science.149.3685.754

[47] Flotte T, Carter B, Conrad C, Guggino W, Reynolds T, Rosenstein B, et al. A phase I study of an adeno-associated virus-CFTR gene vector in adult CF patients with mild lung disease. Hum Gene Ther. 1996;7:1145–59. 8773517 10.1089/hum.1996.7.9-1145

[48] Zaiss AK, Liu Q, Bowen GP, Wong NC, Bartlett JS, Muruve DA. Differential activation of innate immune responses by adenovirus and adeno-associated virus vectors. J Virol. 2002;76:4580–90. 11932423 10.1128/JVI.76.9.4580-4590.2002 PMC155101

[49] Pillay S, Zou W, Cheng F, Puschnik AS, Meyer NL, Ganaie SS, et al. Adeno-associated Virus (AAV) Serotypes Have Distinctive Interactions with Domains of the Cellular AAV Receptor. J Virol. 2017;91:e00391–17. 28679762 10.1128/JVI.00391-17 PMC5571256

[50] Hulliger EC, Hostettler SM, Kleinlogel S. Empowering retinal gene therapy with a specific promoter for human rod and cone ON-bipolar cells. Mol Ther Methods Clin Dev. 2020;17:505–19. 32258214 10.1016/j.omtm.2020.03.003 PMC7114634

[51] Nieuwenhuis B, Laperrousaz E, Tribble JR, Verhaagen J, Fawcett JW, Martin KR, et al. Improving adeno-associated viral (AAV) vector-mediated transgene expression in retinal ganglion cells: comparison of five promoters. Gene Ther. 2023;30:503–19. 36635457 10.1038/s41434-022-00380-z PMC10284706

[52] Kiss S, Oresic Bender K, Grishanin RN, Hanna KM, Nieves JD, Sharma P, et al. Long-term safety evaluation of continuous intraocular delivery of aflibercept by the intravitreal gene therapy candidate ADVM-022 in nonhuman primates. Transl Vis Sci Technol. 2021;10:34. 33532145 10.1167/tvst.10.1.34 PMC7846953

[53] de Smet MD, Lynch JL, Dejneka NS, Keane M, Khan IJ. A subretinal cell delivery method via suprachoroidal access in minipigs: safety and surgical outcomes. Invest Ophthalmol Vis Sci. 2018;59:311–20. 29346488 10.1167/iovs.17-22233

[54] Yiu G, Chung SH, Mollhoff IN, Nguyen UT, Thomasy SM, Yoo J, et al. Suprachoroidal and subretinal injections of AAV using transscleral microneedles for retinal gene delivery in nonhuman primates. Mol Ther Methods Clin Dev. 2020;16:179–91. 32055646 10.1016/j.omtm.2020.01.002 PMC7005511

[55] Shen J, Kim J, Tzeng SY, Ding K, Hafiz Z, Long D, et al. Suprachoroidal gene transfer with nonviral nanoparticles. Sci Adv. 2020;6:eaba1606. 32937452 10.1126/sciadv.aba1606 PMC7458446

[56] Yin D, Ling S, Wang D, Dai Y, Jiang H, Zhou X, et al. Targeting herpes simplex virus with CRISPR-Cas9 cures herpetic stromal keratitis in mice. Nat Biotechnol. 2021;39:567–77. 33432198 10.1038/s41587-020-00781-8 PMC7611178

[57] Uehara H, Zhang X, Pereira F, Narendran S, Choi S, Bhuvanagiri S, et al. Start codon disruption with CRISPR/Cas9 prevents murine Fuchs’ endothelial corneal dystrophy. Elife. 2021;10:e55637. 34100716 10.7554/eLife.55637 PMC8216720

[58] Heath Jeffery RC, Mukhtar SA, McAllister IL, Morgan WH, Mackey DA, Chen FK. Inherited retinal diseases are the most common cause of blindness in the working-age population in Australia. Ophthalmic Genet. 2021;42:431–9. 33939573 10.1080/13816810.2021.1913610 PMC8315212

[59] Liew G, Michaelides M, Bunce C. A comparison of the causes of blindness certifications in England and Wales in working age adults (16-64 years), 1999–2000 with 2009–2010. BMJ Open. 2014;4:e004015. 24525390 10.1136/bmjopen-2013-004015 PMC3927710

[60] Schofield D, Kraindler J, Tan O, Shrestha RN, West S, Hart N, et al. The health care and societal costs of inherited retinal diseases in Australia: a microsimulation modelling study. Med J Aust. 2023;219:70–6. 37301731 10.5694/mja2.51997 PMC10952471

[61] Schneider N, Sundaresan Y, Gopalakrishnan P, Beryozkin A, Hanany M, Levanon EY, et al. Inherited retinal diseases: Linking genes, disease-causing variants, and relevant therapeutic modalities. Prog Retin Eye Res. 2022;89:101029. 34839010 10.1016/j.preteyeres.2021.101029

[62] Kumaran N, Moore AT, Weleber RG, Michaelides M. Leber congenital amaurosis/early-onset severe retinal dystrophy: clinical features, molecular genetics and therapeutic interventions. Br J Ophthalmol. 2017;101:1147–54. 28689169 10.1136/bjophthalmol-2016-309975 PMC5574398

[63] den Hollander AI, Roepman R, Koenekoop RK, Cremers FP. Leber congenital amaurosis: genes, proteins and disease mechanisms. Prog Retin Eye Res. 2008;27:391–419. 18632300 10.1016/j.preteyeres.2008.05.003

[64] Jo DH, Song DW, Cho CS, Kim UG, Lee KJ, Lee K, et al. CRISPR-Cas9-mediated therapeutic editing of Rpe65 ameliorates the disease phenotypes in a mouse model of Leber congenital amaurosis. Sci Adv. 2019;5:eaax1210. 31692906 10.1126/sciadv.aax1210 PMC6821465

[65] Suh S, Choi EH, Leinonen H, Foik AT, Newby GA, Yeh WH, et al. Restoration of visual function in adult mice with an inherited retinal disease via adenine base editing. Nat Biomed Eng. 2021;5:169–78. 33077938 10.1038/s41551-020-00632-6 PMC7885272

[66] Jang H, Jo DH, Cho CS, Shin JH, Seo JH, Yu G, et al. Application of prime editing to the correction of mutations and phenotypes in adult mice with liver and eye diseases. Nat Biomed Eng. 2022;6:181–94. 34446856 10.1038/s41551-021-00788-9

[67] She K, Liu Y, Zhao Q, Jin X, Yang Y, Su J, et al. Dual-AAV split prime editor corrects the mutation and phenotype in mice with inherited retinal degeneration. Signal Transduct Target Ther. 2023;8:57. 36740702 10.1038/s41392-022-01234-1 PMC9899767

[68] Verbakel SK, van Huet RAC, Boon CJF, den Hollander AI, Collin RWJ, Klaver CCW, et al. Non-syndromic retinitis pigmentosa. Prog Retin Eye Res. 2018;66:157–86. 29597005 10.1016/j.preteyeres.2018.03.005

[69] Wilson JH, Wensel TG. The nature of dominant mutations of rhodopsin and implications for gene therapy. Mol Neurobiol. 2003;28:149–58. 14576453 10.1385/MN:28:2:149

[70] Bakondi B, Lv W, Lu B, Jones MK, Tsai Y, Kim KJ, et al. In vivo CRISPR/Cas9 gene editing corrects retinal dystrophy in the S334ter-3 rat model of autosomal dominant retinitis pigmentosa. Mol Ther. 2016;24:556–63. 26666451 10.1038/mt.2015.220 PMC4786918

[71] Qin H, Zhang W, Zhang S, Feng Y, Xu W, Qi J, et al. Vision rescue via unconstrained in vivo prime editing in degenerating neural retinas. J Exp Med. 2023;220:e20220776. 36930174 10.1084/jem.20220776 PMC10037108

[72] Tanna P, Strauss RW, Fujinami K, Michaelides M. Stargardt disease: clinical features, molecular genetics, animal models and therapeutic options. Br J Ophthalmol. 2017;101:25–30. 27491360 10.1136/bjophthalmol-2016-308823 PMC5256119

[73] Stenson PD, Mort M, Ball EV, Chapman M, Evans K, Azevedo L, et al. The Human Gene Mutation Database (HGMD((R))): optimizing its use in a clinical diagnostic or research setting. Hum Genet. 2020;139:1197–207. 32596782 10.1007/s00439-020-02199-3 PMC7497289

[74] Trapani I, Toriello E, de Simone S, Colella P, Iodice C, Polishchuk EV, et al. Improved dual AAV vectors with reduced expression of truncated proteins are safe and effective in the retina of a mouse model of Stargardt disease. Hum Mol Genet. 2015;24:6811–25. 26420842 10.1093/hmg/ddv386 PMC4634381

[75] Siles L, Ruiz-Nogales S, Navines-Ferrer A, Mendez-Vendrell P, Pomares E. Efficient correction of ABCA4 variants by CRISPR-Cas9 in hiPSCs derived from Stargardt disease patients. Mol Ther Nucleic Acids. 2023;32:64–79. 36969552 10.1016/j.omtn.2023.02.032 PMC10034418

[76] Khanani AM, Thomas MJ, Aziz AA, Weng CY, Danzig CJ, Yiu G, et al. Review of gene therapies for age-related macular degeneration. Eye. 2022;36:303–11. 35017696 10.1038/s41433-021-01842-1 PMC8807824

[77] Ciulla T, Pollack JS, Williams DF. Visual acuity outcomes and anti-VEGF therapy intensity in macular oedema due to retinal vein occlusion: a real-world analysis of 15,613 patient eyes. Br J Ophthalmol. 2021;105:1696–704. 33055088 10.1136/bjophthalmol-2020-317337 PMC8639936

[78] Ciulla TA, Hussain RM, Pollack JS, Williams DF. Visual acuity outcomes and anti-vascular endothelial growth factor therapy intensity in neovascular age-related macular degeneration patients: a real-world analysis of 49 485 eyes. Ophthalmol Retin. 2020;4:19–30. 10.1016/j.oret.2019.05.017 31324588

[79] Ciulla TA, Pollack JS, Williams DF. Visual acuity outcomes and anti-VEGF therapy intensity in diabetic macular oedema: a real-world analysis of 28 658 patient eyes. Br J Ophthalmol. 2021;105:216–21. 32265201 10.1136/bjophthalmol-2020-315933 PMC7848066

[80] A Randomized, Partially Masked, Controlled, Phase 2b/3 Clinical Study to Evaluate the Efficacy and Safety of RGX-314 Gene Therapy in Participants With nAMD (ATMOSPHERE) ClinicalTrials.gov identifier: NCT04704921. Updated 2023-05-22. Accessed 1st Decmber 2023 https://clinicaltrials.gov/study/NCT04704921?term=ATMOSPHERE&rank=1.

[81] A Phase 2, Randomized, Dose-escalation, Ranibizumab-controlled Study to Evaluate the Efficacy, Safety, and Tolerability of RGX-314 Gene Therapy Delivered Via One or Two Suprachoroidal Space (SCS) Injections in Participants With Neovascular Age-Related Macular Degeneration (nAMD) (AAVIATE) ClinicalTrials.gov identifier: NCT04514653. Updated 2023-05-22. Accessed 1st December 2023 https://clinicaltrials.gov/study/NCT04704921?term=ATMOSPHERE&rank=1.

[82] A Phase 2, Randomized, Dose-escalation, Observation-controlled Study to Evaluate the Efficacy, Safety, and Tolerability of RGX-314 Gene Therapy Delivered Via a Single Suprachoroidal Space (SCS) Injections in Participants With Diabetic Retinopathy (DR) Without Center Involved-Diabetic Macular Edema (CI-DME)(ALTITUDE) ClinicalTrials.gov identifier: NCT04567550. Updated 2023-05-22. Accessed 1st December 2023 https://clinicaltrials.gov/study/NCT04567550?term=ALTITUDE&intr=diabetic%20retinopathy&rank=1

[83] A Phase I/IIa (Phase 1/Phase 2a), Open-label, Multiple-cohort, Dose-escalation Study to Evaluate the Safety and Tolerability of Gene Therapy With RGX-314 in Subjects With Neovascular AMD (nAMD) ClinicalTrials.gov identifier: NCT03066258. Updated 2023-05-16. Accessed 1st December 2023 https://clinicaltrials.gov/study/NCT03066258?term=NCT03066258&rank=1.

[84] Busbee B, Boyer DS, Khanani AM, Wykoff CC, Pieramici DJ, Regillo C, et al. Phase 1 study of intravitreal gene therapy with ADVM-022 for neovascular AMD (OPTIC TRAIL). Invest Ophth Vis Sci. 2021;62:352.

[85] Bahadorani S, Singer M. Recent advances in the management and understanding of macular degeneration. F1000Res. 2017;6:519. 28491291 10.12688/f1000research.10998.1 PMC5399962

[86] Dreismann AK, McClements ME, Barnard AR, Orhan E, Hughes JP, Lachmann PJ, et al. Functional expression of complement factor I following AAV-mediated gene delivery in the retina of mice and human cells. Gene Ther. 2021;28:265–76. 33750925 10.1038/s41434-021-00239-9 PMC8149295

[87] FOCUS: An Open Label First in Human Phase I/II Multicentre Study to Evaluate the Safety, Dose Response and Efficacy of GT005 Administered as a Single Subretinal Injection in Subjects With Macular Atrophy Due to AMD ClinicalTrials.gov identifier: NCT03846193. Updated 2023-12-21. Accessed 21st December 2023 https://clinicaltrials.gov/study/NCT03846193?term=NCT03846193&rank=1

[88] EXPLORE: A Phase II, Outcomes Assessor-masked, Multicentre, Randomised Study to Evaluate the Safety and Efficacy of Two Doses of GT005 Administered as a Single Subretinal Injection in Subjects With Geographic Atrophy Secondary to Age-related Macular Degeneration ClinicalTrials.gov identifier: NCT04437368. Updated 2023-12-21. Accessed 21st December 2023 https://clinicaltrials.gov/study/NCT04437368?cond=NCT04437368&term=NCT04437368&rank=1.

[89] HORIZON: A Phase II, Open-label, Outcomes-assessor Masked, Multicentre, Randomised, Controlled Study to Evaluate the Safety and Efficacy of Two Doses of GT005 Administered as a Single Subretinal Injection in Subjects With Geographic Atrophy Secondary to Dry Age-related Macular Degeneration ClinicalTrials.gov identifier: NCT04566445. Updated 2023-12-19. Accessed 21st December 2023 https://clinicaltrials.gov/study/NCT04437368?cond=NCT04437368&term=NCT04437368&rank=1.

[90] Holz F Phase 1 Study of JNJ-81201887 Gene Therapy in Geographic Atrophy (GA) Due to Age-related Macular Degeneration (AMD). EURETINA; Amsterdam 2023. 10.1016/j.ophtha.2024.06.013 38909914

[91] Lad EM, Chao DL, Pepio A, Zhang W, Capuano G, Rogers A, et al. Pooled safety analysis of a single intravitreal injection of JNJ-1887 (gene therapy, AAVCAGsCD59) in patients with age-related macular degeneration (AMD). Invest Ophth Vis Sci. 2023;64.

[92] A Study to Evaluate Intravitreal JNJ-81201887 (AAVCAGsCD59) Compared to Sham Procedure for the Treatment of Geographic Atrophy (GA) Secondary to Age-related Macular Degeneration (AMD) ClinicalTrials.gov identifier: NCT05811351. Updated 2023-12-19. Accessed https://clinicaltrials.gov/study/NCT04437368?cond=NCT04437368&term=NCT04437368&rank=1.

[93] Tham YC, Li X, Wong TY, Quigley HA, Aung T, Cheng CY. Global prevalence of glaucoma and projections of glaucoma burden through 2040: a systematic review and meta-analysis. Ophthalmology. 2014;121:2081–90. 24974815 10.1016/j.ophtha.2014.05.013

[94] Han X, Gharahkhani P, Hamel AR, Ong JS, Renteria ME, Mehta P, et al. Large-scale multitrait genome-wide association analyses identify hundreds of glaucoma risk loci. Nat Genet. 2023;55:1116–25. 37386247 10.1038/s41588-023-01428-5 PMC10335935

[95] Maihofner C, Schlotzer-Schrehardt U, Guhring H, Zeilhofer HU, Naumann GO, Pahl A, et al. Expression of cyclooxygenase-1 and -2 in normal and glaucomatous human eyes. Invest Ophthalmol Vis Sci. 2001;42:2616–24. 11581208

[96] Chern KJ, Nettesheim ER, Reid CA, Li NW, Marcoe GJ, Lipinski DM. Prostaglandin-based rAAV-mediated glaucoma gene therapy in Brown Norway rats. Commun Biol. 2022;5:1169. 36329259 10.1038/s42003-022-04134-w PMC9633612

[97] Wu J, Bell OH, Copland DA, Young A, Pooley JR, Maswood R, et al. Gene therapy for glaucoma by ciliary body aquaporin 1 disruption using CRISPR-Cas9. Mol Ther. 2020;28:820–9. 31981492 10.1016/j.ymthe.2019.12.012 PMC7054720

[98] Martinez T, Gonzalez MV, Roehl I, Wright N, Paneda C, Jimenez AI. In vitro and in vivo efficacy of SYL040012, a novel siRNA compound for treatment of glaucoma. Mol Ther. 2014;22:81–91. 24025749 10.1038/mt.2013.216 PMC3978804

[99] Kim MH, Lim SH. Matrix metalloproteinases and glaucoma. Biomolecules. 2022;12:1368. 36291577 10.3390/biom12101368 PMC9599265

[100] O’Callaghan J, Crosbie DE, Cassidy PS, Sherwood JM, Flugel-Koch C, Lutjen-Drecoll E, et al. Therapeutic potential of AAV-mediated MMP-3 secretion from corneal endothelium in treating glaucoma. Hum Mol Genet. 2017;26:1230–46. 28158775 10.1093/hmg/ddx028 PMC5390678

[101] Gerometta R, Spiga MG, Borras T, Candia OA. Treatment of sheep steroid-induced ocular hypertension with a glucocorticoid-inducible MMP1 gene therapy virus. Invest Ophthalmol Vis Sci. 2010;51:3042–8. 20089869 10.1167/iovs.09-4920 PMC2891463

[102] Stone EM, Fingert JH, Alward WL, Nguyen TD, Polansky JR, Sunden SL, et al. Identification of a gene that causes primary open angle glaucoma. Science. 1997;275:668–70. 9005853 10.1126/science.275.5300.668

[103] Fingert JH, Stone EM, Sheffield VC, Alward WL. Myocilin glaucoma. Surv Ophthalmol. 2002;47:547–61. 12504739 10.1016/s0039-6257(02)00353-3

[104] Jain A, Zode G, Kasetti RB, Ran FA, Yan W, Sharma TP, et al. CRISPR-Cas9-based treatment of myocilin-associated glaucoma. Proc Natl Acad Sci USA. 2017;114:11199–204. 28973933 10.1073/pnas.1706193114 PMC5651749

[105] Ozcan AA, Ozdemir N, Canataroglu A. The aqueous levels of TGF-beta2 in patients with glaucoma. Int Ophthalmol. 2004;25:19–22. 15085971 10.1023/b:inte.0000018524.48581.79

[106] Pena JD, Taylor AW, Ricard CS, Vidal I, Hernandez MR. Transforming growth factor beta isoforms in human optic nerve heads. Br J Ophthalmol. 1999;83:209–18. 10396201 10.1136/bjo.83.2.209 PMC1722920

[107] Rayana NP, Sugali CK, Dai J, Peng M, Liu S, Zhang Y, et al. Using CRISPR interference as a therapeutic approach to treat TGFbeta2-induced ocular hypertension and glaucoma. Invest Ophthalmol Vis Sci. 2021;62:7. 34499703 10.1167/iovs.62.12.7 PMC8434756

[108] Gupta V, You Y, Li J, Gupta V, Golzan M, Klistorner A, et al. BDNF impairment is associated with age-related changes in the inner retina and exacerbates experimental glaucoma. Biochim Biophys Acta. 2014;1842:1567–78. 24942931 10.1016/j.bbadis.2014.05.026

[109] Pease ME, McKinnon SJ, Quigley HA, Kerrigan-Baumrind LA, Zack DJ. Obstructed axonal transport of BDNF and its receptor TrkB in experimental glaucoma. Invest Ophthalmol Vis Sci. 2000;41:764–74. 10711692

[110] Chen H, Weber AJ. BDNF enhances retinal ganglion cell survival in cats with optic nerve damage. Invest Ophthalmol Vis Sci. 2001;42:966–74. 11274073

[111] Domenici L, Origlia N, Falsini B, Cerri E, Barloscio D, Fabiani C, et al. Rescue of retinal function by BDNF in a mouse model of glaucoma. PLoS ONE. 2014;9:e115579. 25536045 10.1371/journal.pone.0115579 PMC4275209

[112] Martin KR, Quigley HA, Zack DJ, Levkovitch-Verbin H, Kielczewski J, Valenta D, et al. Gene therapy with brain-derived neurotrophic factor as a protection: retinal ganglion cells in a rat glaucoma model. Invest Ophthalmol Vis Sci. 2003;44:4357–65. 14507880 10.1167/iovs.02-1332

[113] Di Polo A, Aigner LJ, Dunn RJ, Bray GM, Aguayo AJ. Prolonged delivery of brain-derived neurotrophic factor by adenovirus-infected Muller cells temporarily rescues injured retinal ganglion cells. Proc Natl Acad Sci USA. 1998;95:3978–83. 9520478 10.1073/pnas.95.7.3978 PMC19948

[114] Frank L, Ventimiglia R, Anderson K, Lindsay RM, Rudge JS. BDNF down-regulates neurotrophin responsiveness, TrkB protein and TrkB mRNA levels in cultured rat hippocampal neurons. Eur J Neurosci. 1996;8:1220–30. 8752592 10.1111/j.1460-9568.1996.tb01290.x

[115] Osborne A, Khatib TZ, Songra L, Barber AC, Hall K, Kong GYX, et al. Neuroprotection of retinal ganglion cells by a novel gene therapy construct that achieves sustained enhancement of brain-derived neurotrophic factor/tropomyosin-related kinase receptor-B signaling. Cell Death Dis. 2018;9:1007. 30258047 10.1038/s41419-018-1041-8 PMC6158290

[116] Khatib TZ, Osborne A, Yang S, Ali Z, Jia W, Manyakin I, et al. Receptor-ligand supplementation via a self-cleaving 2A peptide-based gene therapy promotes CNS axonal transport with functional recovery. Sci Adv. 2021;7:eabd2590. 33789891 10.1126/sciadv.abd2590 PMC8011959

[117] Nishijima E, Honda S, Kitamura Y, Namekata K, Kimura A, Guo X, et al. Vision protection and robust axon regeneration in glaucoma models by membrane-associated Trk receptors. Mol Ther. 2023;31:810–24. 36463402 10.1016/j.ymthe.2022.11.018 PMC10014229

[118] Bradke F, Marin O. Editorial overview: development and regeneration: nervous system development and regeneration. Curr Opin Neurobiol. 2014;27:iv–vi. 24929993 10.1016/j.conb.2014.05.007

[119] Petrova V, Pearson CS, Ching J, Tribble JR, Solano AG, Yang Y, et al. Protrudin functions from the endoplasmic reticulum to support axon regeneration in the adult CNS. Nat Commun. 2020;11:5614. 33154382 10.1038/s41467-020-19436-y PMC7645621

[120] Li B, Tan W, Wang Z, Zhou H, Zou J, Li Y, et al. Progress and prospects of gene therapy in ophthalmology from 2000 to 2022: A bibliometric analysis. Heliyon. 2023;9:e18228. 37539253 10.1016/j.heliyon.2023.e18228 PMC10395483

